# Harm reduction through housing first: an assessment of the Emergency Warming Centre in Inuvik, Canada

**DOI:** 10.1186/s12954-016-0128-8

**Published:** 2017-02-07

**Authors:** Michael G. Young, Kathleen Manion

**Affiliations:** School of Humanitarian Studies, Royal Roads University, Victoria, BC V9B 5Y2 Canada

**Keywords:** Harm reduction, Homelessness, Concurrent disorders, Housing first, Aboriginal, Qualitative methods

## Abstract

**Background:**

This research examines the effectiveness of an Emergency Warming Centre (EWC) in Inuvik, Canada, at reducing rates of morbidity and mortality for homeless persons with concurrent disorders (mental health problems and addictions). Inuvik is a small town of approximately 3500 residents, with over 65% being Aboriginal. The town is situated on the Beaufort Delta in the Western Canadian Arctic and is subject to oil and gas extraction-based boom and bust economic cycles. The centre provided food and accommodation for those under the influence of alcohol or drugs who had no other place to stay.

**Methods:**

Qualitative interviews about users’ experiences at the centre were conducted with guests, as they were called, centre staff and other key stakeholders in autumn 2014 and spring 2015. Samples of (9) respondents and (7) stakeholders provided significant information about the importance of the EWC. The content of the qualitative data with guests and stakeholders were analyzed for emergent themes.

**Results:**

Several emergent themes and subthemes related to participants’ experiences at the EWC and success of the centre. Overall, the results showed that guests benefitted from a safe place to stay and felt better about their overall health.

**Conclusions:**

Compared with research on wet shelters in New Zealand, Great Britain and the US, this research reveals that harm reduction-based models for homeless persons with concurrent disorders require significant investments in infrastructure, which are not readily available. Yet, the lessons learned from these jurisdictions might be extrapolated to communities like Inuvik to develop alternative housing strategies.

## Background

While homelessness in developed nations is documented historically, its significance as a social problem gained significant attention in the 1980s [15]. Since then, homelessness has grown to be a perplexing and vexatious problem for policy makers, and an embarrassment to nations and communities. Within the Canadian context, homelessness is at crisis levels [7]. While urban homelessness may have been the impetus for attention, one that initially occupied the foreground of research and policy development, attention to rural homelessness emerged as its own significant problem in the 1990s [29]. Rural homelessness may share key elements with its urban counterpart, but it remains on the periphery of attention as it contradicts common conceptions of health and social well-being often associated with life in rural communities. Significantly, rurality may actually add to and exacerbate the problem of homelessness in terms of health and well-being. This is particularly true in the Canadian Arctic, where life is either viewed as uninhabitable or viewed as communal, mutually supportive and in harmony with nature [35, 43, 44]. Arguably, neither image is accurate, but the rurality and remoteness of the area coupled with the extreme weather mean that there are fewer services to draw on, more acute housing needs and increased difficulty of bringing in supplies including food and building material. This exacerbates issues of homelessness as availability of affordable housing generally cannot keep pace with demand.

To be sure, the causes of homelessness are numerous, complex and not easily solved. Substance abuse, mental illness, physical and emotional abuse, marital breakdown, loss of employment, transition from institutionalized care, and economic factors such as, lack of affordable and/or available housing, and economic restructuring figure prominently in research on homelessness [10, 27]. Some authors have explored socio-structural explanations and found that the demise of social housing programmes in Canada in the 1990s is clearly linked to a surge in homeless populations, both urban and rural [5, 7, 34]. Similarly, in the UK, May et al. [26] trace the growth in numbers of the single persons experiencing homelessness through the 1980s and 1990s with the dismantling of the welfare state and then with the increasing governmentality of neo-liberal and social interventionist policies to tackle chronic street homeless in the 1990s and early 2000s. Similarly, Milbourne and Cloke [30] later suggest that the last few decades have seen increasing complexity emerge in homelessness in Australia as the demographics of those experiencing homeless shift to encompass a wider variety of people impacted by socio-economic changes in health, housing and poverty. In New Zealand, issues of street homelessness have been relatively hidden. In comparing Auckland in New Zealand to Vancouver and Edmonton in Canada, Collins [7] highlights the significant difference in welfare and housing policies of the two countries. Where Canada experienced a significant increase in homelessness in the wake of cutbacks to social housing in the early 1990s and early 2000s, Collins’ [7] research suggests that New Zealand has been somewhat sheltered from homelessness because of social housing together with cultural practices that protect socially excluded family members. However, more recent neo-liberal policies and increasing housing cost may render a different picture in the near future, as intimated by Anderson and Collins [2] in a comparative study exploring indigenous homelessness between Canada, New Zealand, and Australia.

Addressing large scale socio-structural challenges in a global economic context could possibly be one of the most difficult challenges for developed nations in recent history. Neo-liberal policies related to housing crises and the demise of social welfare policies have clearly taxed communities and individuals [24] leading to an expanding population of hard to house people, those who are chronically, cyclically, temporarily or transitionally homeless [10]. The global economic collapse in 2008 propelled homelessness to new levels as nations scrambled to balance budgets through austerity measures which negatively affected social welfare policies [12, 15, 32].

Given the range of causal factors, it is not surprising that effective solutions to homelessness are few, and when developed, difficult to implement. Addressing large scale socio-structural challenges while simultaneously dealing with individual problems like addiction, mental health and other personal problems are daunting tasks indeed. One possible approach that has witnessed some success is called “Housing First”. In essence,

Housing First involves providing clients with assistance in finding and obtaining safe, secure and permanent housing as quickly as possible. Key to the Housing First philosophy is that individuals and families are not required to first demonstrate that they are ‘ready’ for housing. Housing is not conditional on sobriety or abstinence [16].

Housing First is grounded in a harm reduction philosophy. It is an approach that assumes helping people from where they currently live rather than from artificial, unrealizable and agency-derived goals of abstinence and sobriety results in better long-term results [17].

Research supports the application of Housing First models in urban and rural Canadian contexts [18, 23, 41], and in Australia, Great Britain, New Zealand and USA [28], particularly for homeless persons dealing with substance abuse issues. While context may dictate subtle variations, harm reduction principles stipulate that safe, stable housing, client centred support, highly integrated care teams, such as assertive case management, and transitional/supportive housing options lead to lower levels of service consumption, higher levels of treatment seeking behaviour, lower levels of substance abuse and stable housing over the long term [23, 28, 41].

This paper details the findings from an evaluation of the effectiveness of the Emergency Warming Centre (EWC) in Inuvik during the winter months in 2014–2015. This harm reduction project, loosely based on a Housing First model, provides the context for exploring comparative approaches for addressing homelessness and concurrent disorders in rural areas.

### Research context

Situated in the Beaufort Delta, the town of Inuvik is the largest community in the Western Canadian Arctic. The population of Inuvik has remained relatively stable, at roughly 3400 since the early 21st century. Roughly two thirds of the town’s population are Aboriginal, largely Gwich’in and Inuvialuit [22]. Inuvik is unique in that it was a planned community developed by the Canadian Federal Government and meant to serve as a beacon for sovereignty, eventually housing the largest military installation in the Canadian north [9]. However, its role as a military outpost lost significance with the discovery of rich oil and natural gas deposits in the 1970s [4, 11]. The advent of oil and gas exploration brought with it a cycle of economic booms and busts with resource extraction industries taking a centre stage in economic development with companies vying for market dominance. The proposed construction of the Mackenzie Valley pipeline became a hotly debated project, for two primary reasons. First, acrimonious relationships between original inhabitants of the Mackenzie Valley and southern business interests led to the Berger [4] report which recommended that Aboriginal land claims be settled before oil and gas exploration commenced. Second, the estimated high cost of oil and gas extraction delayed construction of the pipeline, and when combined with market volatility, stymied further extraction research projects by oil and gas companies [1, 8, 11].

The impact of resource extraction is associated with economic boom and bust cycles and the ongoing problems associated with the experiences of Aboriginal peoples. The frontier character accompanying oil and gas exploration brought with it substantial social impacts, the effects of which are still present. While the causal linkages between homelessness and concurrent disorders are debated at many levels, a substantial body of research in northern Canada identifies the negative impact of colonization, resource extraction and economic development on Aboriginal peoples [1, 3, 8, 11, 37, 38]. Following Berger [4] comments, research on communities affected by resource extraction repeatedly shows that boom cycles are associated with (a) increasing crime and addiction rates, (b) housing shortages and increased housing costs and (c) strains on public services including health, social work and most levels of government infrastructure [3, 13, 37–39].

Starting in the early 1990s, community groups in Inuvik recognized the emergence of a growing number of visible homeless persons, which has resulted in an overwhelming demand for shelter [19–21, 42]. This population is frequently referred to as chronic or long-term homeless, but this definition belies other elements of true homelessness. Hard to house populations are comprised of persons whom may be temporarily homeless, cyclically homeless or simply in transition from being housed to hard to house [10]. The transition shelter established in the 1990s is unable to accommodate the number of potential clients and does not accept hard to house persons under the influence of drugs or alcohol. Consequently, those hard to house persons unable to access the transition shelter are left to their own devices in terms of finding accommodation. Most are unable to stay with family or friends because they have “worn out their welcome” with problem behaviours. In the past, some have stayed in the RCMP cells. However, the mandate of the RCMP does not include housing leaving the agency at risk of being cited for policy violations (e.g. unlawful confinement). Others manage to sleep under buildings or in larger sections of the “utilidor”, an above ground utility carrying service. However, the risk of serious illness, injury or death is a concern in colder months of the year for those not able to access adequate accommodation [20, 21, 43, 44].

### Purpose

This research examines the effectiveness of the EWC from October 2014 to May 2015 in Inuvik, Canada. This evaluation was situated within a wider research context that asked what role substance abuse and mental health issues play in individual’s pathways into and out of homelessness. Built on the foundations of previous research looking at rural homelessness in the Beaufort Delta by Young and Moses [43], this research focused more specifically on the effectiveness of the pilot project that emerged to redress issues of homelessness and concurrent disorders in Inuvik through the EWC. With strong initial community support in Inuvik, the Inuvik Interagency Committee initiated the pilot programme in the autumn of 2013. Housed within the Anglican Church, the pilot was further expanded and reintroduced in October 2014 running through to May 2015. The centre opened nightly from 7 pm to 9 am. It provided a safe, warm place to sleep, and dinner and breakfast. Drinking and substance use was prohibited in the centre. However, unlike the permanent homeless shelter in Inuvik, the EWC was accessible to people who were under the influence of drugs or alcohol.

The primary objective of the centre was to keep the homeless population from dying of exposure. Secondary objectives of the centre were to increase the access to supports for users of the centre and to improve their health and social well-being by providing stability in diet and warm sleeping quarters. The purpose of the evaluation was to assess the effectiveness of the EWC in terms of the improvements in the lives of homeless persons with concurrent disorders in Inuvik. Specifically, the evaluation questioned the efficacy of EWC’s ability to improve the health and social functioning of its users by providing stable dietary intake and safe warm sleeping arrangements.

## Methods

The research took a fundamentally community-based research approach working with the local community [31, 33] employing a participatory action research (PAR) methodology. The researchers obtained ethical review from the Royal Roads Research Ethics Board, and approval for the research in the Northwest Territories was granted by the Aurora Research Institute. Initially, the research involved qualitative and quantitative research; however, the small sample rendered the results of quantitative aspect of the research inconclusive. Consequently, this paper presents the findings from the qualitative component of the research. Respondents were recruited to participate by the researchers who visited the EWC shortly after opening in October 2014. A sample of nine out of a possible 20 “guests”, as they were called, completed 1 h interviews and participated in focus groups at two points in time: October 2014 and April 2015. Interviews with two additional guests were completed at either the pre- or post-period, but they were left out of the analysis as they did not complete both of the interviews or focus groups. Guests were asked about their health, well-being, lifestyle and support systems, their thoughts on the impact of the centre and their perspectives on the community and community-based supports. While an interview guide was used to start questioning, probes were used to encourage guests to elaborate their answers (see Appendixes 1 and 2 for the April 2015 interviews schedules with guests and stakeholders). This approach allowed for more authentic, rich and arguably more accurate responses from guests [25]. Three interviewers were used in each round, with two remaining consistent. Each guest was given a $25 gift card for completing the October component and another $25 gift card for the April aspect of the research, which were redeemable at a local store.

In addition to guests, key stakeholders of the EWC, including volunteers, staff members, board members and founding members, participated in focus groups and 1-h-long interviews in October and April. Stakeholders were contacted in August 2014 and asked if they would be willing to support and participate in the research. The interviews and focus groups with stakeholders were aided by an interview guide with questions on the management and functioning of the centre and how attendance at the centre affected guests. The interview guides were created and validated by the research team using findings of previous research done in the rural homelessness and housing first initiatives. In total, seven interviews were held with staff and key stakeholders. Four focus groups were held, with between four and 12 participants. The same themes formed the basis of questions asked, but these followed a more emergent path. Participants included a mixture of guests, staff, board members, founding members and other interested community members.

Situated within a symbolic interaction framework, which highlights the intersubjective nature of social reality [36], a social constructivist approach to research guided that data analysis. Homeless persons with concurrent disorders are marginalized from the broader community, but they represent a distinct community unto themselves. Accordingly, the data analysis lends itself to an interpretive framing which emphasizes equality. Harris [14] argues that social constructionists are compelled to engage in research that can improve equality of relations and social condition. Thus, the content analysis of the interview and focus group data are informed by interpretive, analytical frames that provide participants an expressive voice [25] with a view to promote social change. Based on the constructivist approach used here, the content analysis from the interview and focus group data with guests and stakeholders were used to identify emergent themes.

## Results

The results provided some specific information regarding the effectiveness of the Emergency Warming Shelter in Inuvik, including suggestions for future endeavours. This section summarizes key findings before outlining the emergent themes and subthemes. For Inuvik, this and previous research indicate there is a clear migration of homeless men and women into Inuvik from surrounding areas. Earlier research by Young and Moses [43] supports the claim that services for this population are fragmented, inadequate or ill prepared to cope with the special needs of this population. It supports the observation that mental health and addiction issues are common within the population experiencing homelessness and that mental health issues are often undiagnosed and/or untreated. This is partially due to a lack of service. Similarly, there is a severe lack of supportive housing designed to support a population with specific and often numerous issues. For example, there is an evidence of multiple intersections of violence and trauma, including post-traumatic stress disorder, intergenerational trauma and experience of residential schools, which exacerbate the potential for mental health and addiction issues.

### Emergent themes

Rich information was gathered from the qualitative data. While the findings were not necessarily representative, they painted a picture of life for the respondents and other service users of the EWC. The themes and subthemes that emerged were categorized as (1) typical day, (2) use and appreciation of centre, (3) obstacles to recovery (including “resiliency, physical and mental health of guests”), (4) substance use, (5) housing and other services needed,(6) dearth of professional support and accessing help, (7) sense of connection, (8) challenges and successes at the centre, (9) vision, policies and roles, (10) sense in change in service provision and future ideas and (11) experience of homelessness in Inuvik.

Interviews with stakeholders and guests suggested that overall alcohol or drug consumption for most guests declined with their attendance at the centre. In addition to interviews, the superintendent of the RCMP was asked to share data regarding changes noted by police during the operation of the EWC. Notably, the superintendent reported a decrease in the number of admissions to the cells over the period of EWC operation and thus a corresponding decrease in charges against the guests of the centre.

#### Typical day

As the shelter is only open overnight, days for guests are long. Despite their generally upbeat attitudes, they also painted a picture of boredom and disenfranchisement. The majority of respondents said that they spent most of their days walking the streets or shorelines of the Mackenzie River. Some suggested they spent some time with their family or friends, on the streets or in their homes. Most respondents suggested that they spent time drinking each day. Some respondents had casual work or family obligations in which to attend. Alternatively, a few mentioned that they visited either the library or the soup kitchen during part of the day. One respondent said he used the recreation complex to shower. At night, those who did not stay at the centre slept in tents, cubby holes, under buildings, in utilidors and sometimes with friends or family members or in bush camps during the warmer months. On rare occasions, respondents suggested they stayed at either in the other homeless shelter or the women’s shelter, the hospital or the police station lockup. The pattern did not change significantly between interview periods, although several did suggest in the second period they spent time waiting for the EWC to open.

#### Use and appreciation of the centre

##### Use of the centre

There was a continuum of use of the shelter. Some guests used it nightly, where others used it occasionally when other options ran out (for instance if they were kicked out by family or by the other shelter).

##### Appreciation of the centre

Guests reported a high level of appreciation for the provision of the service. The main reasons respondents gave for appreciating the EWC included the following: allowing them to stay independent and “not to cause a nuisance” to friends and family, providing them with a warm, safe, dry place to sleep and a place to store their belongings and not freezing to death. They also appreciated having someone to talk to and not being watched. For some, it also offered a sense of hope for things to improve. One guest suggested:

Well this warming shelter is good, I do like the fact that it is here to help people cuz in Inuvik it’s very harsh… it’s a good thing that this is here. You know like people aren’t sleeping under buildings freezing to death.

Another guest said “They can’t shut it down…they are great guys here.” Another guest reflected:

I feel a lot better because you know I know I am here and I am safe and you know… so it’s really difficult to you know everyday try to struggle to you know keep your spirits up…

#### Obstacles to recovery

Staff and other stakeholders identified a number of barriers to recovery for guests. Their list was slightly different than guests. For them, key issues included lack of identification for travel for guests and more fundamentally, a lack of local services, coupled with either a duplication of services or a lack of service coordination in town.

Obstacles identified by guests have been grouped in five subthemes–resiliency, physical and mental health issues; substance use; housing and other services; lack of professional support and accessing help.

### Resiliency, physical and mental health

Overall, respondents demonstrated a high degree of resiliency. They reported significant levels of physical health issues, ranging from chronic to acute illness and injuries, but these issues were minimized by the guests themselves. A guest suggested “nothing in my body is working right”. Similarly, although most reported having no mental health issues or current problems, a number of respondents disclosed multiple traumatic experiences and significant losses. A number of experiences of psychological and physical violence were shared. One guest spoke about experiencing violence in the past to the degree that it “tortured me enough to live on painkillers”. Despite this observation, most described their mental health as good. However, this may relate more to their fear of stigma or to a lack of knowledge than to their mental health.

### Substance use

According to the staff and other stakeholders, most of the guests were alcohol users (usually Sherry). Some occasionally used marijuana, but other substance use was uncommon. Towards the end of the period, there were some groups that appeared to be using crystal meth.

A guest claimed that alcohol was used as self-medication so extensively that it had become chronic. “For the past couple of years, a typical day is looking for my next drink.” Another guest suggested:

I am an alcoholic, it’s an addiction. Right, and like I said it’s my choice whether I want to or not and if you had other support though, maybe you wouldn’t so much that’s oh that’s a factor of boredom also, there’s nothing to do.

While guests did not identify any problems with other substances, opinions were split on whether they believed the EWC could or would impact their drinking. Some said it would reduce or regularize their drinking and make it safer, but others felt it would not make a difference on levels of consumption. Most respondents had optimistic but realistic perspectives on their ability to stop drinking.

### Housing and other services

According to respondents, two main themes emerged when looking at obstacles to housing and general recovery. One was the lack of accessible or permanent housing, and the other was alcohol addiction. The housing insecurity that respondents felt included not only a lack of access to basic shelter but also to warmth, food and storage. It also involved a lack of respite from boredom, disrupted sleep, a lack of access to facilities to maintain basic hygiene and insecurity of belongings. The EWC offered some respite to these areas, but not to all of them. For instance, respondents suggested that even with the support of the EWC, they had nowhere to store their personal belongings during the day, nowhere to go in the day and no access to wash themselves or their clothes.

There was scant evidence that respondents were drawing on many services. However, most suggested that they had applied for housing and were on a waiting list. One guest said “I wish I had my place, if I could find a place, I could try to get my life back together.”

Some noted that they used the soup kitchen and, occasionally, the hospital. A few mentioned that they had gone to counselling in the past but were not currently doing so. Some noted they had previously gone to detoxification treatment or substance abuse counselling but were not currently accessing these. A few noted they occasionally accessed Alcoholics Anonymous meetings or church support. Only one respondent noted the need to seek legal help. A few respondents occasionally worked casually, although one respondent appeared to work more regularly.

### Dearth of professional support

Respondents noted that they needed more access to appropriate counselling, housing support, detoxification, a programme on the land and support for obtaining identification (which is difficult to do with no fixed abode). A few noted that they had been encouraged to go back to school, but none suggested that they were currently attending classes. One respondent said:

They say oh you should go back to school but it is not easy being homeless and try to go back to school. It’s not easy trying to get a job and not have anything to eat or anywhere to wash your clothes or have a shower or you know. That’s tough.

According to the staff and other stakeholders, there were gaps in services both within the centre and across the town. They highlighted activities during the day, bathing and clothes washing facilities; community based supports; access to rehabilitation and mental health services as the most critical gaps.

### Accessing help

Although some services exist in the community, according to the staff and other stakeholders, the guests reported both having little access to services, either by choice or availability and rarely utilising services that exist. Staff and other stakeholders suggested that once accessing a service, guests had a difficult time using the services consistently for a variety of reasons.

In answer to “do you access any services in town”, one guest suggested “NO, I’m pretty much lost in the dark”. Another suggested “No, I just live day by day by myself. Work when I can for the day”.

Some did not bother to access services, or they were on waitlists or they used them and found they were not appropriate. Almost all suggested they were on a housing waitlist. The most common source of support was found with family or social networks, but anecdotally, this appeared more common for females than males. A couple of guests suggested that they used the centre staff for support. Overall, the picture for accessing formal or informal support was haphazard.

#### Social support and connectedness

In terms of social support, respondents were mixed. For the most part, female respondents were more likely to suggest they had good social support networks with family and/or friends and that they both gave and received support from others.

Analysis of guest’s statements illustrates that a sense of connection to family, friends and Aboriginal band was mixed. Most suggested they felt reasonably connected to the centre but noted that they had experienced prejudice within the wider community. One respondent suggested:

it’s frustrating like to live in you know you set up a tent and make your little spot somewhere and someone comes along and destroys it, ignorant kids or ignorant people doing that. I don’t know. I never caught anyone destroying my stuff before. Pretty sneaky.

Another stated “they see you as a lowlife alcoholic… you are still a human being…you still have feelings”. Despite this, respondents generally felt a connection to Inuvik.

#### Challenges and successes at the centre

Several behaviour issues emerged that required good policy and practice. The prominent concerning behaviour was lack of respect for some centre staff and an occasional violent disruption towards the latter half of operation. A high turnover of staff, board members and management escalated this issue and did not support an environment where problems were dealt with consistently. One guest suggested:

There’s also the um, conflict of people coming drunk and causing trouble and you know…, it’s tough. You know cuz, cuz they, they allow it. But, nobody wants to put up with somebody that’s cranky when they are drunk. All they do is constantly run their mouth.

Staff and other stakeholders identified key systemic issues that limited the efficacy of the centre. These included turnover of staff in wider health and social service systems in town, lack of comparative information on rural and northern homelessness and appropriate interventions, inconsistent commitment from, and reliability of, staff, visible community support for the centre including from the church, insufficient training for staff and staff expertise, lack of transition system out of homelessness, staff, volunteer and board turnover, and varying commitment from the Inter-Agency Committee.

Overall, the key success of the centre was that none of the guests died while using the centre. Staff and other stakeholders accounted for the success for the centre by highlighting good practice, such as strong managerial support for staff, celebrating the examples of success of some guests, consistency in the availability of the centre and strong support from police and ambulance services.

#### Vision, policy and roles

##### Vision

Stakeholders illustrated a common understanding of the overarching vision and purpose of the centre, i.e. to ensure survival of guests by offering a safe place to sleep and some food. Similarly, they expressed a clear idea of the target population, chronic to transitional homeless with addiction issues. In short, one staff member suggested the centre “is just aimed at being that safe place for people even if they are intoxicated”. Another stakeholder suggested the purpose was “to keep people alive and this has been successful”.

##### Policy

Staff and other stakeholders suggested that the development of policies for the EWC was slow in coming. This was complicated by a high level of turnover by staff and board members. Initially, attempts were made to bring some policies in, for instance a “no violence” policy, while other policies such as washing laundry and cleaning responsibilities developed with time and experience. Another example that was trialled was a token system to promote prosocial behaviour, but this was never fully implemented. In reflecting on what was going well, a staff member suggested:

We know what to expect, and by keeping it consistent from night to night, it just allows us to build that rapport with the clients because there are no surprises. And they know the drill.

##### Roles

The staff, management and board members outlined some of the key roles of staff. These included the ability to set boundaries, enforce rules, supervise guests, manage intake, clean the centre and prepare food. In thinking about what the centre could be, visions included an increase of services offered and better integration with services in Inuvik. They also saw the need for a new location and an expansion to include transitioning support.

#### Sense of change in service provision and future ideas

Respondents reported that little had changed in Inuvik in terms of services, other than the introduction of the EWC. They offered a number of ideas for services or supports that could help the community experiencing homelessness to move to independence. These included “someone to talk to”, “a place to live”, help in accessing housing, support with obtaining identification, “warmth”, “a laundromat”, work, detox and a graduated wet-dry shelter that moved people to independence.

In exploring options for future iterations of the centre, staff and other stakeholders suggested that they could explore using an external agency with experience in the field to manage the centre and to find a new and more appropriate space for the centre that allowed longer hours of operation. Other focus areas included exploring the possibility of charging for bed nights and continuing to build community support for the centre.

#### Experience of homelessness in Inuvik

Respondents to the interview demonstrated their tenacity, resiliency and survival instincts. Although a few noted a lack of compassion they had experienced in the town, they were grateful for the support they had experienced from the EWC. One guest suggested that they felt that their opinion did not matter and that it was perceived as uniformed.

Respondents articulated the complexity of the homelessness situation in Inuvik. When asked, what could be done to help, one guest illustrated how challenging it is to find solutions by suggesting:

I couldn’t tell you. To tell you the truth I think about it and I really don’t know. Couldn’t tell you. Like I think about it and it just seems like…

Other guests identified some of the barriers to accessing permanent housing, issues with battling substance abuse and the elemental challenges posed by living in the Far North.

## Discussion

The evaluation of the EWC in Inuvik provides evidence of the potential efficacy of the Housing First wet shelter model for homeless persons with concurrent disorders. As a logical extension of harm reduction approaches to addiction, discussions with guests of the centre suggested they intended to reduce alcohol consumption or had already started taking steps to reduce alcohol intake. Arguably, the EWC had an overall positive effect on many guests in terms of changing alcohol consumption behaviour. Findings from the research suggest guests’ levels of social functioning improved over the pre- and post-test interviews. However, there was a high level of anxiety given the impending closure of the EWC. There were indications that the centre provided a level of habituation, which may have left guests more vulnerable during the period when it was not open.

From guests’ perspectives, the qualitative data provide an encompassing interpretation of the EWC. The importance of the centre for health and overall well-being was a constant theme. A safe and warm place to stay that provided some meals, a place to store one’s personal belongings, shower and laundry facilities provided respite from the life of being homeless and addicted. Connecting guests with health and social services available in the community was not in the centre’s mandate, and few guests accessed resources. Clearly, the results demonstrate a need for more comprehensive, coordinated and inclusive services. This was reiterated by data collected from the centre staff. Respondents relayed that some basic structures were in place to support the guests and staff, but these were relatively rudimentary. In time, these policies and processes could be further developed, including staff training.

The data provide some insights into the effectiveness of the Housing First models in rural and/or northern contexts. Research from several jurisdictions underscores these observations. Regarding harm reduction strategies in Canada, Krause et al. [23] observe that Housing First approaches are correlated with improved health outcomes, reductions in substance use, increased health seeking behaviours and more prosocial activities. Waegemakers Schiff and Turner’s [41] research on rural homelessness in Canada echoes these findings. Moreover, MacIntyre’s [28] research on the effectiveness of 11 wet shelters, those not requiring sobriety, in Canada, England, Ireland, New Zealand and the US, identifies the positive effects of Housing First approaches on clients’ well-being. In all cases, however, Housing First can be considered a necessary but insufficient response to the problems experienced by homeless persons with concurrent disorders. Other necessary ingredients for serving this population include programming that addresses the myriad issues associated with homelessness and concurrent disorders. Housing First programmes based on harm reduction require a client centred approach, intensive case management that is responsive to individual clients’ needs, continuous support for clients, respectful and trained staff, interagency collaboration between service providers and access to community programming and social activities [23, 40, 41]. MacIntyre summarizes this approach succinctly when she states that wet shelters work because “…leaders of organizations providing these services and the staff who, on a daily basis, offer a mix of compassion, realism and professional support to people who desperately in need” (2009, p.3).

## Conclusions and recommendations

This research provided an evaluation of the Inuvik EWC operating between October 2014 and May 2015. The EWC was loosely based on a harm reduction approach as it offered meals and a safe, warm place to sleep for homeless or hard to house persons with concurrent disorders who could not access other sleeping accommodations because they were under the influence of alcohol or drugs, or because of their behaviour. Admittedly, the research does have shortcomings—it is based on a small sample of homeless persons and stakeholders. As well, the centre was not designed to provide any services other than food and nighttime winter shelter, and as such, does not fit a true Housing First model of intervention. In addition, although guests can be inebriated when they enter the centre, they cannot drink on the premises or after they have checked in for the night; therefore, the EWC is not designed to be a wet shelter. These aspects alone should prove fatal to the centre’s operation, yet without the centre, there is a possibility that the guests of the centre would have been at serious risk of illness, injury and death. Although the data provide qualified support for the EWC, it is clear that it served its function of keeping guests safe and alive. That the RCMP reported fewer admissions to their cells is an evidence of a positive effect on the EWC in terms of the appropriate use of police services.

Overall, the results from this research corroborate much of the extant literature on Housing First in term of the elements necessary for successful Housing First approaches to homelessness based on harm reduction. This research established a starting point in rural, northern contexts by identifying the complex interplay of complications between homelessness, mental illness and addiction in harsh and remote environments, particularly in the wake of massive global economic changes affecting resource extraction in northern Canada. This also provides reason to challenge the status quo notion of rural homelessness articulated by Cloke and Milbourne who suggested that “…it remains the case that rurality can also be intertwined with political conservatism, moral individualism and cultural tendencies to blame the victim” ([6], p. 273). A holistic systems approach that recognize and address multiple and intersecting issues that lead to, and keep people within, homelessness are much more effective in terms of long-term strategies. Harm reduction strategies such as wet shelters and the Housing First model offer a more holistic approach, but they come with public criticism. They require clear vision, community consultation, support and education, and strong allied support systems of transition. In addition to continued and larger research projects on homelessness and concurrent disorders in rural locales, future research should examine the most effective strategies used to promote and develop housing first strategies in communities lacking the infrastructure and expertise to implement harm reduction approaches to homelessness and concurrent disorders.

## Appendix 1

### Preliminary evaluation of the emergency warming shelter for homeless persons with concurrent disorders in Inuvik

#### Guests interview

*Tell me a little about yourself and if anything has changed since last time we spoke*

1. Where do you go on a typical day? And how often do you stay in the Centre?
  *Prompt- specifically the library, the store, elsewhere*
2. What problems do you feel you are experiencing right now?
  *Prompt - Do you have concerns about your mental health, physical health, wellbeing, social network?*

*What Supports Do You Have? Has this changed since last time we spoke?*

3. What kind of problems do you seek help for? Who do you go to or where do you go?
4. Do others come to you when they have a problem?
5. What prompts you to seek support? What prompts others to seek your support?
6. What gets in the way of you seeking support/help/seeking treatment? Can you give me an example?
7. What services do you draw on?
  *Prompt - specific where and when (in addition to survey question)*
8. How connected do you feel to: the shelter, your friends, your family; Inuvik, your community/band? Where do you most feel you belong?

*How helpful has the Emergency Warming Centre been? Has this changed since last time we spoke?*

9. Where did you stay at night before the Centre opened? Where did you stay at night last year?
10. Do you think being in the shelter has, or will, change things for you, for instance:
  - Your health or wellbeing (*physical or mental*)
  - Your drinking patterns
  *Prompt - where and when you drink, how much you drink, and with whom you drink?*
  c. Your level of support
  d. Other
11. If the shelter was not here where would you be? What are your options?
12. Has the Centre been helpful for you?
13. Where do you see yourself in the spring?

*Tell me about your experience in Inuvik. Has this changed since last time we spoke?*

14. What services are available for you? What services are needed to help people who are homeless?
15. Tell me about finding shelter/housing in Inuvik.
16. Have you seen any changes recently in the services or housing that are available in Inuvik?

## Appendix 2

### Preliminary evaluation of the emergency warming shelter for homeless persons with concurrent disorders in Inuvik

#### Staff focus group/interviews

These questions were asked in October 2014. I ask that you reflect on them and suggest if anything has changed.

1. In your opinion, what was the rationale behind opening the Emergency Warming Shelter?
2. Has it emerged as it was intended?
3. What is going well so far? What could be improved?
4. What roles are covered by staff? What strengths do the staff bring to the job?
5. Who is using the Centre? Is this the population that was intended to use the Centre?
6. What positive impact has the Emergency Warming Centre had on the residents (e.g. regarding their health, addictions, basic needs, mental health, or social functioning)? Were any impacts unintended?
7. What services are residents calling for? Which services are they accessing most?
8. What are the biggest gaps in service? What is most urgently needed?
9. What would you like to see in the future? (*Within the next six months, within the next few years*)?
10. (New Question) What has been the biggest learning regarding the Centre over the last 6 months? What would you have done differently? What has been a particular success?

## Abbreviations

EWC: Emergency Warming Centre
IIC: Inuvik Interagency Committee
RCMP: Royal Canadian Mounted Police
